# A Comparison of Rule-based Analysis with Regression Methods in Understanding the Risk Factors for Study Withdrawal in a Pediatric Study

**DOI:** 10.1038/srep30828

**Published:** 2016-08-26

**Authors:** Mona Haghighi, Suzanne Bennett Johnson, Xiaoning Qian, Kristian F. Lynch, Kendra Vehik, Shuai Huang, The TEDDY Study Group, Marian Rewers, Katherine Barriga, Judith Baxter, George Eisenbarth, Nicole Frank, Patricia Gesualdo, Michelle Hoffman, Jill Norris, Lisa Ide, Jessie Robinson, Kathleen Waugh, Jin-Xiong She, Desmond Schatz, Diane Hopkins, Leigh Steed, Angela Choate, Katherine Silvis, Meena Shankar, Yi-Hua Huang, Ping Yang, Hong-Jie Wang, Jessica Leggett, Kim English, Richard McIndoe, Angela Dequesada, Michael Haller, Stephen W. Anderson, Anette G. Ziegler, Heike Boerschmann, Ezio Bonifacio, Melanie Bunk, Johannes Försch, Lydia Henneberger, Michael Hummel, Sandra Hummel, Gesa Joslowski, Mathilde Kersting, Annette Knopff, Nadja Kocher, Sibylle Koletzko, Stephanie Krause, Claudia Lauber, Ulrike Mollenhauer, Claudia Peplow, Maren Pflüger, Daniela Pöhlmann, Claudia Ramminger, Sargol Rash-Sur, Roswith Roth, Julia Schenkel, Leonore Thümer, Katja Voit, Christiane Winkler, Marina Zwilling, Olli G. Simell, Kirsti Nanto-Salonen, Jorma Ilonen, Mikael Knip, Riitta Veijola, Tuula Simell, Heikki Hyöty, Suvi M. Virtanen, Carina Kronberg-Kippilä, Maija Torma, Barbara Simell, Eeva Ruohonen, Minna Romo, Elina Mantymaki, Heidi Schroderus, Mia Nyblom, Aino Stenius, Åke Lernmark, Daniel Agardh, Peter Almgren, Eva Andersson, Carin Andrén-Aronsson, Maria Ask, Ulla-Marie Karlsson, Corrado Cilio, Jenny Bremer, Emilie Ericson-Hallström, Thomas Gard, Joanna Gerardsson, Ulrika Gustavsson, Gertie Hansson, Monica Hansen, Susanne Hyberg, Rasmus Håkansson, Sten Ivarsson, Fredrik Johansen, Helena Larsson, Barbro Lernmark, Maria Markan, Theodosia Massadakis, Jessica Melin, Maria Månsson-Martinez, Anita Nilsson, Emma Nilsson, Kobra Rahmati, Sara Rang, Monica Sedig Järvirova, Sara Sibthorpe, Birgitta Sjöberg, Carina Törn, Anne Wallin, Åsa Wimar, William A. Hagopian, Xiang Yan, Michael Killian, Claire Cowen Crouch, Kristen M. Hay, Stephen Ayres, Carissa Adams, Brandi Bratrude, Greer Fowler, Czarina Franco, Carla Hammar, Diana Heaney, Patrick Marcus, Arlene Meyer, Denise Mulenga, Elizabeth Scott, Jennifer Skidmore, Erin Small, Joshua Stabbert, Viktoria Stepitova, Dorothy Becker, Margaret Franciscus, MaryEllen Dalmagro-Elias Smith, Ashi Daftary, Jeffrey P. Krischer, Michael Abbondondolo, Lori Ballard, Rasheedah Brown, David Cuthbertson, Christopher Eberhard, Veena Gowda, Hye-Seung Lee, Shu Liu, Jamie Malloy, Cristina McCarthy, Wendy McLeod, Laura Smith, Stephen Smith, Susan Smith, Ulla Uusitalo, Jimin Yang, Beena Akolkar, Thomas Briese, Henry Erlich, Steve Oberste

**Affiliations:** 1 Department of Industrial and Management Systems Engineering, University of South Florida, Tampa, Florida, USA; 2 Department of Behavioral Sciences and Social Medicine, College of Medicine, Florida State University, Tallahassee, Florida, USA; 3 Department of Electrical and Computer Engineering, Texas A&M University, College Station, Texas, USA; 4 Health Informatics Institute, University of South Florida, Tampa, Florida, USA; 5 Department of Industrial & Systems Engineering, University of Washington, Seattle, Washington, USA; 6 University of Colorado, Anschutz Medical Campus, Barbara Davis Center for Childhood Diabetes, Aurora, Colorado, United States; 7 University of Florida, Gainesville, Florida, United States; 8 Pediatric Endocrine Associates, Atlanta, Georgia, United States; 9 Diabetes Research Institute, Center for Regenerative Therapies, TU Dresden, Institute of Psychology, University of Graz, Austria; 10 Von Hauner Children´s Hospital, Department of Gastroenterology, Ludwig Maximillians University Munich, Germany; 11 Research Institute for Child Nutrition, Dortmund, Germany; 12 University of Turku, Turku, Finland; 13 Turku University Hospital, Hospital District of Southwest Finland, Turku, Finland; 14 University of Tampere, Tempere, Finland; 15 Tampere University Hospital, Tempere, Finland; 16 University of Kuopio, Kuopio, Finland; 17 Oulu University Hospital, Oulu, Finland; 18 University of Oulu, Oulu, Finland; 19 National Institute for Health and Welfare, Helsinki, Finland; 20 Lund University, Lund, Sweden; 21 Pacific Northwest Diabetes Research Institute, Seattle, Washington, United States; 22 Children’s Hospital of Pittsburgh of UPMC, Pittsburgh, Pennsylvania, United States; 23 National Institutes of Diabetes and Digestive and Kidney Diseases, Bethesda, Maryland, United States; 24 Columbia University, New York, United States; 25 Children’s Hospital Oakland Research Institute, California, United States; 26 Centers for Disease Control and Prevention, Atlanta, Georgia, United States.

## Abstract

Regression models are extensively used in many epidemiological studies to understand the linkage between specific outcomes of interest and their risk factors. However, regression models in general examine the average effects of the risk factors and ignore subgroups with different risk profiles. As a result, interventions are often geared towards the average member of the population, without consideration of the special health needs of different subgroups within the population. This paper demonstrates the value of using rule-based analysis methods that can identify subgroups with heterogeneous risk profiles in a population without imposing assumptions on the subgroups or method. The rules define the risk pattern of subsets of individuals by not only considering the interactions between the risk factors but also their ranges. We compared the rule-based analysis results with the results from a logistic regression model in The Environmental Determinants of Diabetes in the Young (TEDDY) study. Both methods detected a similar suite of risk factors, but the rule-based analysis was superior at detecting multiple interactions between the risk factors that characterize the subgroups. A further investigation of the particular characteristics of each subgroup may detect the special health needs of the subgroup and lead to tailored interventions.

Understanding the factors associated with the risk of individuals withdrawing from a study is an important first step towards identifying the eventual health needs of different individuals within a population^1^. This lays the foundation to develop and deliver appropriate resources to the right targets, called “tailored health interventions”. Evidence suggests that individuals prefer tailored care to a standardized care that is designated for the average population^2^,^3^,^4^,^5^. Therefore, health professionals need to identify the subgroups of individuals characterized by different patterns of risk factors. However, rather than identifying subgroups, traditional intervention studies often focus on identification of risk factors that are associated with the outcome of interest for the population as a whole^1^,^6^,^7^. One commonly adopted approach is to use logistic regression to identify factors associated with study withdrawal^8^,^9^,^10^. However, this approach only models the average effects of the risk factors. Consequently, it is likely that the interventions developed from regression models will be geared toward the average member of the population, with less consideration of the special needs of different subgroups^11^.

The aim of the present study is to illustrate the use of the rule-based analysis^12^,^13^,^14^ as an exploratory technique in an epidemiologic context. The rule-based analysis^12^,^13^,^14^ is particularly useful for identifying the subgroups embedded in a dataset—whose members share similar risk patterns—that influence the outcome of interest. A rule describes the range of values on one or more risk factors that are associated with either an increase or decrease in risk for withdrawal in a subset of individuals. Thus, rules provide a natural semantics to define the risk pattern of subsets of individuals while each rule may indicate a specific unmet health need or warning signal for study withdrawal. By identifying the unknown rules from observational studies, a comprehensive set of risk-predictive rules can be considered as a set of sensors, providing us personalized risk estimation by looking into the risk patterns endorsed by each individual.

Specifically, we used a recently developed rule-discovery algorithm for the rule-based analysis, the RuleFit method^14^, which is one example from a huge array of rule-based methods that are promising for epidemiologic research. The RuleFit method has an advantage over logistic regression because it relies on a nonparametric model with fewer modeling assumptions, random forest^13^, which is capable of identifying the risk predictive rules. There is no need to explicitly include covariate interactions or transformations into the model because of the recursive splitting structure used in generating the random forest. Also, the rule-based analysis permits an individual’s risk to be predicted on the basis of only one, or at most a few, risk factors, whereas scores derived from regression models require that all covariates be available.

We demonstrate the rule-based analysis using data from a large multinational epidemiological natural history study of type 1 diabetes mellitus (T1DM), the Environmental Determinants of Diabetes in the Young (TEDDY) study^15^. Specifically, we use the rule-based analysis for predicting study withdrawal during the first year of the TEDDY study, by effectively integrating the psychosocial, demographic, and behavioral risk factors collected at study inception. We compare the rule-based analysis with a previous analysis that was conducted on the same data^10^. The previous analysis used traditional logistic regression methods to identify factors collected at study inception that were strongly associated with study withdrawal during the first year of TEDDY^10^. However, the way these factors interact with each other and the way these interactions might define subgroups in the study population with different risk levels remain unknown. Therefore, we tested the hypothesis that the rule-based analysis can identify the risk-predictive rules useful for stratifying the study population into different subgroups with different risk levels for study withdrawal in the first year of TEDDY. The previous analysis^10^ provided us an opportunity for critically evaluating the potential added value of a rule-based analysis over that provided by traditional logistic regression methods. Also, we considered how the rule-based method could lead to more informed intervention strategies or prioritization of the intervention allocation to the study participants. By conducting this comparison, we also hoped to identify some practical guidelines for when we should use rule-based methods and when regressions model would be more preferable, enriching the analytic toolbox of today’s epidemiologists to address the emerging data challenges.

## Materials and Methods

### The TEDDY study

TEDDY is a natural history study that seeks to identify the environmental triggers of autoimmunity and T1DM onset in genetically at-risk children identified at three centers in the United States (Colorado, Washington, and Georgia/Florida) and three centers in Europe (Finland, Germany, and Sweden). Infants from the general population with no immediate family history of T1DM, as well as infants who have a first degree relative with T1DM, are screened for genetic risk at birth using human leukocyte antigen genotyping. Parents with infants at increased genetic risk for T1DM are invited to participate in TEDDY. Parents are fully informed of the child’s increased genetic risk and the protocol requirements of the TEDDY study, including the requirement that eligible infants must join TEDDY before the infant is 4.5 months of age. The TEDDY protocol is demanding with study visits for blood draws and other data and sample collection scheduled every three months during the first four years of the child’s life and biannually thereafter. Parents are also asked to keep detailed records of the child’s diet, illnesses, life stresses and other environmental exposures. TEDDY obtains written consent from the parents shortly after child’s birth for obtaining genetic and other samples from the infant and also parents. Detailed study design and methods have been previously published^15^. The study methods have been carried out in accordance with the approved guidelines by local Institutional Review or Ethics Boards and monitored by an External Evaluation Committee formed by the National Institutes of Health. The experimental protocols of the study were approved by the National Institute of Health.

### Study sample

This analysis focused on two groups of families from the general population used in the previous logistic regression study^10^: 2,994 families who had been active in TEDDY for ≥1 year and 763 families who withdrew from TEDDY during the first year. Both the prior and current analyses were limited to general population families because study withdrawal among the first degree relatives population was rare.

### Study variables

Study variables were selected from data collected on the screening form at the time of the child’s birth and from interview and questionnaire data collected at the baby’s first TEDDY visit. These variables included: demographic characteristics—TEDDY country (Finland, Germany, Sweden, United States); mother’s age (in years); child’s gender; maternal health during pregnancy‒number of illnesses, gestational diabetes or type 2 diabetes (yes/no); mother’s lifestyle behaviors during pregnancy—smoked at any time during pregnancy (yes/no), alcohol consumption (no alcohol, 1–2 times per month, ≥3 times per month during each trimester), employment status (worked during all 3 trimesters/did not work at all or reduced work hours); baby’s health status‒birth complications (yes/no), health problems since birth (yes/no), hospitalizations after birth (yes/no); number of stressful life events during and after pregnancy; mother’s emotional status including worry and sadness during pregnancy (rated on 5 point scales), anxiety about the child’s risk of developing diabetes measured by a six-item scale adapted from the State component of the State-Trait Anxiety Inventory^2^,^3^,^4^; the accuracy of the mother’s perception of the child’s risk for developing diabetes (accurate: indicating the child’s T1DM risk was higher or much higher than other children’s T1DM risk; inaccurate: indicating the child’s T1DM risk was the same, somewhat lower or much lower than other children’s T1DM risk); and whether the child’s father completed the initial study questionnaire (yes/no).

### Previous logistic regression results

Multiple logistic regression models were used to identify significant predictors of early withdrawal from TEDDY. Variables were entered in blocks in the following order: demographic variables (country of residence, child’s gender, mother’s age); pregnancy/birth variables (maternal diabetes, illness in mother or child, birth complications, maternal smoking; maternal drinking; maternal employment outside the home, maternal worry or sadness during pregnancy, number of stressful life events occurring during pregnancy or after the child’s birth); father’s participation in TEDDY defined by father’s completion of a brief questionnaire; and mother’s reactions to the baby’s increased T1DM risk (anxiety and accuracy of mother’s perception of the child’s T1DM risk). Nine percent of the study sample (N = 326) had missing data on one or more variables. As expected, those subjects who had difficulty in complying with all data collection (35%) were more likely to withdraw than those with high data collection compliance (19%). While it is unknown what is the underlying mechanism that could explain this association, we suspect that this could indicate that the percentage of missing data is a good indicator that suggests a need for TEDDY study to better communicate with participant families and remove any possible difficulties for them to participate in the study. The analysis was first completed for those with no missing data and then rerun for the full sample using multiple imputation methods to generate appropriate parameter estimates for missing data using the Proc MI and Proc MIANALYZE procedures available from SAS 9.1^5^. Table 1 provides the results of the final logistic regression model for the sample of 3,431 TEDDY participants with no missing data. The model was highly significant (Chi-Square  = 264.87 (12), p < 0001) and accurately placed 81.6% of the sample into their respective group (Actives versus Withdrawals). The data in Table 1 also provides the final logistic regression model for the total sample, with multiple imputation methods used to replace missing data. Because the early withdrawal rate was higher among participants with missing data, we added a variable to the imputed model, >1 missing data point (yes/no). The presence of >1 missing data points predicted early drop-out over and above all other variables in the model. The descriptive information for each of the significant predictors is provided in Table 2.

### Statistical methods

#### Basic idea of the RuleFit method

We use RuleFit^14^ to discover the hidden rules that may be predictive of the risk of early withdrawal in subsets of TEDDY individuals. A rule consists of several interacting risk factors and their ranges. We are interested in the rules by which the subjects can be stratified by distinct risk levels. For example, a rule consisting of State Anxiety Inventory Score >45 and Dad Participation = NO would be useful if the subjects who can be characterized by this rule have a higher risk of early withdrawal. RuleFit is a computational algorithm that can scale up for high-dimensional applications (e.g., with a large number of variables) for rule discovery, which is capable of exhaustively searching for potential rules on a large number of candidate risk factors. It has two phases, the “rule generation phase” and “rule pruning phase”.

#### Rule generation

At this stage, random forest^13^ is used to exhaustively search for candidate rules over the potential risk factors. Random forest is a high-dimensional rule discovery approach that extends traditional decision tree models^12^. Specifically, a random forest estimates a number of trees, with each tree being estimated on a relatively homogenous subpopulation generated by bootstrapping the original dataset. Since each tree employs a set of rules to characterize a subpopulation, the random forest is actually a comprehensive collection of rules that are able to characterize the whole dataset.

#### Rule pruning

As a heuristic and exhaustive search approach, the random forest may produce a large number of rules that can be redundant or irrelevant to predicting early withdrawal due to overfitting. To address this, the sparse regression model^16^,^17^ can be applied to select a minimum set of risk-predictive rules, by using all the potential rules as predictors and the withdrawal status as the outcome. The sparse regression model is a high-dimensional variable selection model that can be applied on a large number of variables, and has been widely used in bioinformatics and systems biology^18^,^19^.

In what follows, we illustrate the details of how the RuleFit method uses the three models, the decision tree, random forest, and sparse linear regression models, in the rule generation stage and the rule pruning stage:

#### Stage 1 of RuleFit - Rule generation

**Figure 1:**
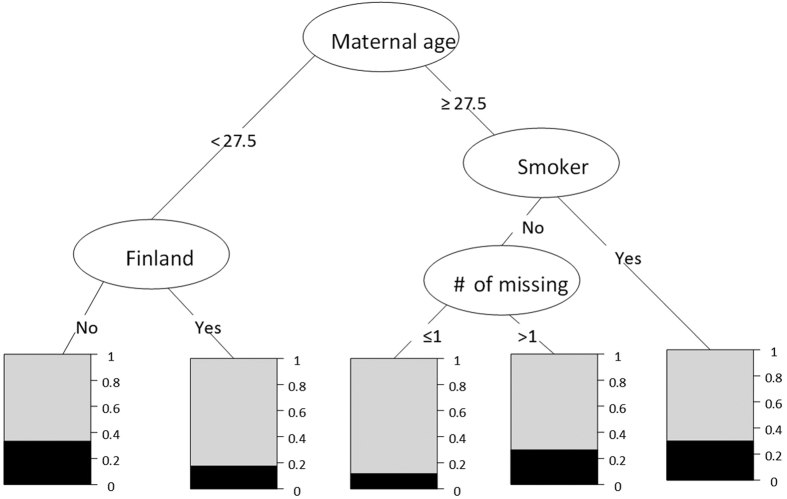
A decision tree learned from the TEDDY data.

Rule generation is computationally challenging, since the number of potential rules grows exponentially in relationship to the number of risk factors. Given such a large number of potential rules, an intelligent rule generator is needed to narrow down the search by effectively detecting high-quality risk-predictive rules. Decision tree learning method provides such an intelligent rule generator. A decision tree is a technique for segmenting the population into different subgroups using a set of rules. For example, we use the decision tree model for analyzing the TEDDY dataset to divide the population into homogeneous subgroups based on the percentage of study withdrawals in each subgroup. The decision tree model is a nonparametric method that automatically explores the given risk factors and their interactions for a tree that has high accuracy in predicting study withdrawal. In our analysis, as shown in Fig. 1, three subgroups with distinct risk levels are identified and can be characterized by rules defined by maternal age, smoking status, number of missing data, and a geographical indicator for Finland. For example, the leftmost node characterizes a subgroup of subjects, in which all of them have Maternal age <27.5 and Finland = NO. The risk of study withdraw in this subgroup is 0.38. This analysis demonstrates that the decision tree model is a powerful tool for detecting the subgroups that can be characterized by rules. Note that, the cut-off value of each factor used in Fig. 1 is automatically determined by the Recursive Partitioning Algorithm (RPA).

One limitation of the decision tree is that only exclusive rules can be identified. For instance, the decision tree in Fig. 1 implies that each participant can only be characterized by one single rule, which doesn’t consider the possibility that a participant may have multiple risk patterns characterized by different factors or different interactions between factors. As a remedy, random forest^13^ is a high-dimensional rule discovery approach that extends traditional decision tree models. It estimates a number of trees: in each iteration, we estimate a decision tree on a bootstrapped sample of the training set, and this process iterates until the pre-specified number of trees is achieved^13^.

To understand the random forest, it is worth mentioning that the essence of this iterative procedure is to generate a large number of substantially different trees, since the more similar the trees are, the less advantage estimating multiple trees has. In order to achieve this goal, randomization methods are used, which is the reason for the name “random forest”. Specifically, in estimating each tree, the bootstrap technique is used for generating a different training sample by randomly reweighting the original dataset. Subsequently, in the estimation of each tree, a subset of risk factors is randomly selected for estimating the tree. Therefore, as each tree is built for a sub-population using a subset of risk factors, the heterogeneity of the participants is well addressed in the random forest model, increasing the likelihood of detecting meaningful risk-predictive rules for different subgroups^13^. As each tree can be decomposed to a number of rules, e.g., in Fig. 1, we could extract at least five rules while each rule corresponds to a leaf node in the tree, with random forest we could collect many rules.

#### Stage 2 of RuleFit - Rule pruning

Rule pruning is essentially a procedure of selecting a subset of rules out of a pool of *q* candidate rules, denoted as *R* = [*R*_1_, *R*_2_, …, *R*_*q*_], which are predictive to the output variable *Y*. This problem is particularly challenging in high-dimensional settings where we have a large number of generated rules and *q* is large. One solution to select the most critical rules is to adopt the *Least Absolute Shrinkage Selection Operator* (LASSO)^16^, which is a sparse linear regression model that is capable of identifying a subset of relevant variables out of a huge list of candidate variables. Specifically, the formulation of LASSO is


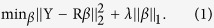


Here, the square error term, 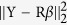 is used to measure the model fit. The L1-norm penalty term ||***β***||_1_ (16), defined as the sum of the absolute values of all elements in *β*, is used to measure the complexity of the regression model. The user-specified penalty parameter, *λ*, aims to achieve an optimal balance between the model fitness and model complexity – larger *λ* will result in sparser estimate for *β*. It has been shown that LASSO is consistent on variable selection both from theoretical research^16^ and empirical studies^17^,^18^,^19^,^20^. Efficient algorithms have been developed to solve the optimization problem, such as the shooting algorithm^16^, proximal gradient algorithms^17^, etc. Through LASSO, we expect that the rules with critical risk factor patterns will be identified with controlled redundancy. In our study, since the output variable *Y*, i.e., the withdrawal status, is a binary variable, the sparse logistic regression^17^ is a better choice than linear regression, which can be readily implemented in the R package of RuleFit^14^.

**Figure 2:**
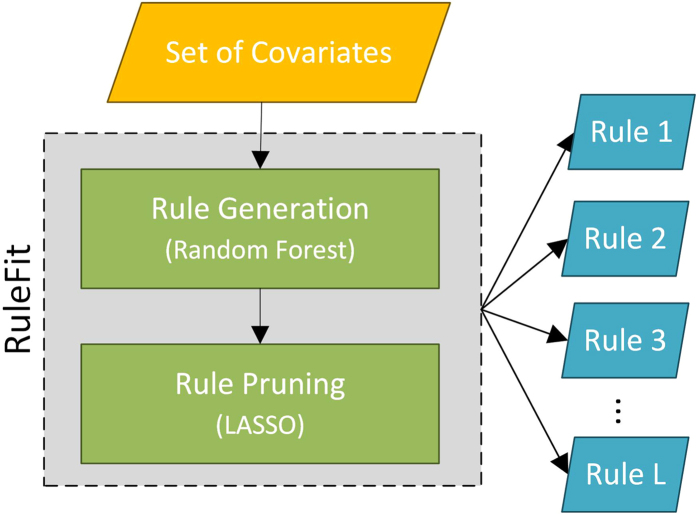
Flow diagram of the RuleFit algorithm.

In summary, RuleFit is computationally efficient since efficient algorithms have been developed for both Random Forest and sparse linear regression models. RuleFit has an automated cross-validation procedure for tuning its parameters, such as the number of trees, the size of the trees and the penalty parameter *λ* in LASSO, which can be used to obtain a set of high-quality rules. More details about RuleFit can be found in^14^. Figure 2 also provides a schematic description of the Rulefit algorithm.

## Results

### Identified risk-predictive rules

Table 3 provides the risk-predictive rules identified by the RuleFit algorithm for the Active and Withdrawn families used in the previous logistic regression analysis^10^. The risk factors identified in the risk-predictive rules are the same as those identified in the previous logistic regression analysis: demographic factors including maternal age and country, maternal lifestyle factors during pregnancy including as smoking, drinking, and working outside the home, psychosocial factors including the mother’s perception of the child’s risk and her anxiety about the child’s risk, dad participation, and the number of missing data points. In addition, the interaction between the state anxiety inventory score with the risk perception accuracy found in the previous study, which further validated in the rule-based analysis (see Table 1 and Table 3). However, the rule-based analysis was more powerful at detecting the interactions between the risk factors. In addition, the rule-based approach identified the number of negative life events as a risk factor, a variable that was not significant in the prior logistic regression analysis. And the rule-based approach found no significant role for child gender, which had a weak effect in the prior analysis (see Table 3). Note that the rules shown in Table 3 were identified by LASSO from more than 2000 candidate rules generated by random forest.

### Investigation of the risk levels of endorsing the risk patterns

**Figure 3:**
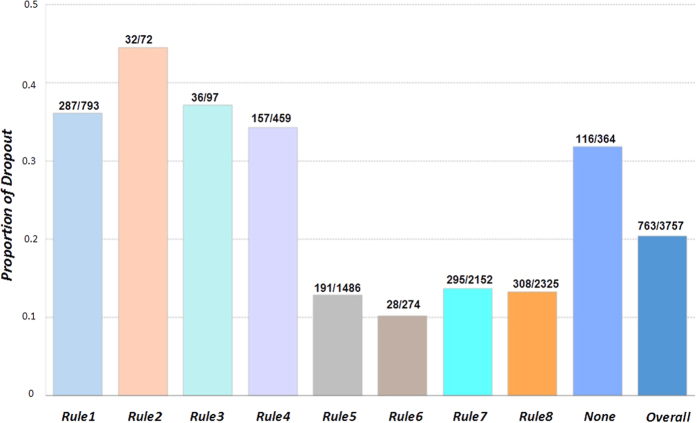
Proportion of early withdrawal of the eight rules and the overall population.

We next investigated the risk level of endorsing each of the rules by computing the study withdrawal rate for each subgroup that endorsed a rule. Figure 3 illustrates the withdrawal rates of each of the eight identified rules as well as the overall withdrawal rate of the whole study population. The number of subjects in each subgroup is also shown in the figure. It is clear that endorsing any of the first four rules will boost the risk of early withdrawal dramatically, while endorsing any of the later four rules will help decrease the risk significantly. Approximately 10 percent of the study population did not fall into any subgroup and their withdrawal rates were relatively high. It could be possible that there are other important subgroups that were not detectable with the available measures. It could also be possible that for this small group the RuleFit is not powerful enough to detect any significant rules, indicating the need for more powerful rule methods. Moreover, it is possible that because the dropout mechanism could be very complicated and involves many aspects such as socioeconomic and psychological factors, the existence of this subgroup indicates a certain level of unpredictability for some cases.

### Investigation of the redundancy of the rules

**Figure 4:**
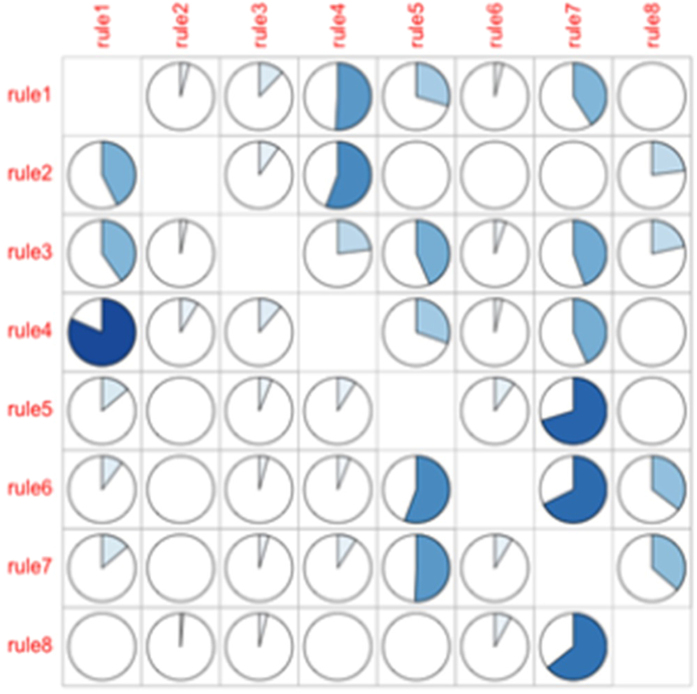
Investigation of the redundancy of the 8 rules. The pie graph on row (corresponds to rule) and column (corresponds to rule j) records the proportion of the participants endorsing rule i who also endorse rule.

One important technical issue in the rule-based analysis is the control of redundancy of the rules. Two rules are redundant if a participant endorses one rule, this participant will endorse the other rule. Obviously, it is less desirable to have two rules that largely overlap with each other. We investigated the redundancy of the 8 rules and presented the results in Fig. 4. Figure 4 can be read in this way: the pie graph on row *i* (corresponds to rule *i*) and column *j* (corresponds to rule *j*) records the proportion of the participants endorsing rule i who also endorse rule j. It can be seen that, the overall redundancy of the rules is slight, although there are some correlations between some rules, such as rule 1 and rule 4, rule 5 and rule 7. The reason for a correlation between two rules may be that both rules share some common risk factors, e.g., both rule 1 and rule 4 involve maternal age < 27.5 in their definitions.

## Discussion

In this article, the rule-base analysis^14^ has been proposed to enrich the toolbox of epidemiological intervention studies that have been relying on regression models. We used data from the TEDDY study and demonstrated that the rule-based analysis can effectively identify risk-predictive rules from the psychosocial, demographic, and behavioral risk factors. The 8 identified rules are found predictive of early withdrawal during the first year of the TEDDY study. The 8 rules involve different sets of risk factors, highlighting the different nature of the withdrawal risk for each of these subgroups. Note that these 8 rules are not exclusive, giving the flexibility that an individual can show multiple risk patterns simultaneously.

We also compared the rule-based analysis with the previous analysis that was conducted on the same data^10^. We found that both methods detected almost the same suite of risk factors, providing validation of our rule-base analysis. Note that the previous analysis only identified the average effects of these risk factors across the whole population, without considering how these risk factors interact with each other in determining the risk of early withdrawal. Although it identified the interaction between the mother’s state anxiety inventory score and her risk perception accuracy, many other interactions remained undetected. The rule-based analysis was superior at detecting multiple interactions between the risk factors, in addition to the interaction between the mother’s state anxiety inventory score and her risk perception accuracy.

As each rule characterizes a distinct risk pattern that consists of different risk factors, a further investigation of the particular characteristics of each rule may help identify the special health needs of the subgroup whose members endorse this rule, leading to tailored interventions. For example, as revealed in rule 3, for mothers who are highly anxious about their child’s T1D risk with a state anxiety inventory score >45, the lack of participation of the father increases the risk of study withdrawal. In an effort to tailor an intervention to this specific subgroup, a study nurse might be assigned to the family having this risk pattern to enhance the psychological support for the mother and encourage the participation of the father. On the other hand, the rules are also helpful for developing general-purpose interventions. For instance, as smoking during pregnancy was important in multiple rules, investigations may be conducted to understand why this behavior is related to the risk of study withdrawal. If smoking during pregnancy was found to be an indicator of less health-conscious attitudes, a tailored intervention might be developed for mothers who smoked during pregnancy to increase their health consciousness in an effort to reduce her risk of study withdrawal. As tailored interventions are developed and deployed, it is also important to evaluate the efficacy of these interventions for the subgroups separately, in order to identify the best intervention strategy for each subgroup.

The rule-based analysis also identified the negative life events as a risk factor of the early withdrawal, which was not detected by the logistic regression model used in the previous study^10^. Previous studies have linked negative life events with immune system functioning^21^,^22^ and the onset of T1DM^23^,^24^. While the mechanism underlying the linkage between the negative life events and study withdrawal remains unknown, it is reasonable to expect that mothers experiencing numerous negative life stresses may not have the personal resources to remain in the study. Certainly tailoring an intervention to this subgroup of individuals seems warranted.

The rule-based method has a number of advantages when handling complex datasets. It can be used with a mix of nominal, ordinal, count or continuous variables and it can combine a mixture of variables—demographic, biological, psychological—without interpretation difficulty. Also, as rules are scale independent, data do not need to be standardized. Finally, the rules will permit some individuals to be classified on the basis of only one, or at most a few, risk factors, whereas risk scores derived from regression models require that all the risk factors are available.

There are limitations of the rule-based approach for epidemiologic studies. First, it is not suitable for studying the overall impact of a single independent variable on the outcome variable. This is because a single independent variable may play a role in multiple rules, which results in difficulty to investigate its overall effect on the whole population. Also, domain insight is very important in the identification of the rules using RuleFit. Due to the automatic nature of the rule-based approach, it is tempting to simply enter all the possible candidate variables into the program without justification of which independent variables should be considered. It has been recommended in the literature^25^ that the prior knowledge regarding the relationship between the independent and dependent variables should be incorporated with the rule-based models. One of the reasons the rule-based approach yielded remarkably similar findings to the logistic regression approach in terms of identifying risk factors per se, is that considerable thought was put into variable selection and measurement. Rule-based models should not be used for blind exploration of large data sets and should benefit from domain experts’ supervision. We agree that a general guidance to use machine learning models for analyzing complex dataset such as TEDDY data is that the new tool should be appropriate to the research question. This is actually one main motivation for our study. As with most observational studies, a significant amount of variation exists among TEDDY subjects. Conventional models such as the logistic regression model cannot sufficiently characterize these variations, since logistic regression model essentially aims to characterize the average effects of the risk factors on a homogeneous population. It is reasonable to suspect that TEDDY population consists of a mix of heterogeneous subpopulations while interactions between variables are essential to define and understand these subpopulations. Thus, the main motivation of this study is to demonstrate the utility of the rule-based approach for analyzing complex datasets such as TEDDY data. Moreover, TEDDY data exhibits some other significant challenges to conventional models that the rule-based method can easily handle as we have articulated above.

Through this study, one of our co-authors (who has been a pediatric psychiatrist for many years and led the previous study on the same dataset using logistic regression model^10^) found that the rule-based method could be a valuable new tool to augment conventional hypothesis-driven research, particularly when theory-driven researchers have limited insight or detailed knowledge about the dataset to be analyzed (e.g., this is very likely as contemporary epidemiologists need to analyze datasets with a diverse set of variables that include traditional epidemiological variables as well as genetic variables (such as SNP variants), virus exposures, omics variables, etc.). Together with the fact that the current study has demonstrated that the rule-based approach could identify risk factors that are consistent with the previous hypothesis-driven research, our study implies that, for those complex datasets, the rule-based method could be used to initiate the analysis process to identify unknown but informative patterns from the dataset that may help theory-driven researchers to generate new hypothesis and better formulate their studies. To facilitate this role, we draw the following practical guidance for how to integrate the rule-based analysis methods into the existing epidemiological toolbox. If there is a strong premise that multiple subgroups may exist in the dataset, the rule-based method could be a very useful approach. On the other hand, subgroups may vary from dataset to dataset, and the rules (and the risk factors involved in these subgroups) identified by the rule-based method may vary from dataset to dataset as well. It is important to understand that the rule-based method is a customized method that is tailored for analyzing an individual dataset, so whether or not the results identified from one dataset could be generalized to another dataset depends on the subgroup structure of the new dataset. While flexibility of an analytic method usually comes with risk of overfitting, a customized method also needs customized expertise or solid domain knowledge of the dataset. Finally, rule-based methods can be considered as opportunistic methods that aim to discover positive patterns, but the results identified by rule-based methods are not necessary exclusive. For example, it is possible that there are more rules besides the eight rules identified from TEDDY cohort by the RuleFit.

In summary, we believe that the rule-based approach will be useful in many epidemiologic studies, particularly with heterogeneous populations consisting of subgroups of individuals. The distinct risk factors that define each subgroup could also reflect a different mechanism of withdrawing from the study, leading to development of different intervention strategies. Besides the utility in designing tailored intervention, it can also help with the prioritization of the intervention targets, e.g., we could choose eliminate a particularly high-risk subgroup at the beginning of a clinical study. Note that the RuleFit algorithm introduced here is one example from a huge array of the rule-based methods that are promising for epidemiologic research in general. How to properly adopt them for addressing the increasing analytic challenges in epidemiologic studies will be an important future research topic. Also, we will investigate how to build predictive models based on the discovered rules, and further validate its predictive performance on another validation dataset that is being collected at TEDDY study.

## Additional Information

**How to cite this article**: Haghighi, M. *et al.* A Comparison of Rule-based Analysis with Regression Methods in Understanding the Risk Factors for Study Withdrawal in a Pediatric Study. *Sci. Rep.* **6**, 30828; doi: 10.1038/srep30828 (2016).

## Figures and Tables

**Table 1:** Previous logistic regression results for the sample with no missing data and the total sample with missing data imputed: Variables associated with study withdrawal in the first year of TEDDY. (Reprinted from Johnson, S. B. *et al.*10 with permission from John Wiley and Sons Inc).

Predictor variable		Sample with No Missing Data (N=3431)						Sample with missing data imputed (N = 3757)		
		Estimate	SE	P-value	OR	95% Confidence Interval		**β**	SE	P-value
Intercept		1.126	0.424	0.008				0.982	0.400	0.014
Country	United States	ref						ref		
	Finland	−0.420	0.130	0.001	0.657	0.509	0.848	−0.431	0.123	0.0004
	Germany	0.278	0.222	0.211	1.321	0.854	2.042	0.154	0.218	0.481
	Sweden	−0.342	0.110	0.002	0.711	0.572	0.882	−0.346	0.104	0.002
Child sex female	No	ref								
	Yes	0.160	0.092	0.081	2.316	1.840	2.915	0.217	0.086	0.012
Maternal age (years)		−0.058	0.009	<0.0001	0.944	0.927	0.961	−0.053	0.009	<0.0001
Maternal Lifestyle Behaviors during Pregnancy										
Smoked	No	ref						ref		
	Yes	0.841	0.117	<0.0001	2.318	1.841	2.918	0.803	0.117	<0.0001
Alcohol consumption in last trimester	None	ref								
	1–2 times/month	−0.343	0.148	0.020	0.709	0.531	0.948	−0.280	0.140	0.045
	>2 times/month	−0.424	0.319	0.183	0.654	0.350	1.222	−0.401	0.299	0.180
Worked all trimesters	No	ref						ref		
	Yes	−0.396	0.095	<0.0001	0.673	0.559	0.811	−0.364	0.090	<0.0001
Dad participation	No	ref						ref		
	Yes	−0.569	0.162	0.0005	0.566	0.412	0.778	−0.608	0.146	<0.0001
Risk perception	Underestimate	ref						ref		
	Accurate	−1.257	0.375	0.0008	0.284	0.137	0.593	−1.032	0.354	0.004
State Anxiety Inventory score		0.001	0.006	0.835	1.001	0.989	1.014	0.001	0.006	0.825
State Anxiety Inventory score x risk perception		0.023	0.009	0.011	1.023	1.005	1.041	0.018	0.009	0.039
>1 missing data points								1.321	0.464	0.007

**Table 2:** Characteristics of TEDDY Actives and Withdrawals. (Reprinted from Johnson, S. B. *et al.*10 with permission from John Wiley and Sons Inc).

Characteristic	Actives (n = 2994)	Withdrawals (n = 763)	Total Sample (n = 3757)
Country	N (%)	N (%)	N
Finland	747(84%)	140(16%)	887
Germany	106(75%)	36(25%)	142
Sweden	1052(82%)	231(18%)	1283
United States	1089(75%)	356(25%)	1445
Child sex	N (%)	N (%)	N
Male	1538 (81%)	352 (19%)	1890
Female	1456 (78%)	411 (22%)	1867
Maternal age (years)	M (SD)	M (SD)	M (SD)
	30.8 (5.0)	28.5 (5.7)	30.4(5.2)
Maternal Lifestyle Behaviors During Pregnancy			
Smoking	N (%)	N (%)	N
Smoked	296(63%)	171(37%)	467
Did not smoke	2602(84%)	510(16%)	3112
Data missing	96(54%)	82(46%)	178
Alcohol consumption at 3^rd^ trimester	N (%)	N (%)	N
Alcohol 1-2 times per month	474(87%)	72(13%)	546
Alcohol ≥ 3 time per month	105(89%)	13(11%)	118
No alcohol	2359(79%)	609(21%)	2968
Data missing	56(45%)	69(55%)	125
Employment status	N (%)	N (%)	N
Worked all 3 trimesters	1418(85%)	251(15%)	1669
Reduced work, quit, or did not work at all	1426(77%)	417(23%)	1843
Data missing	150(61%)	95(39%)	245
Dad Participation in TEDDY	N (%)	N (%)	N
Participated	2813(82%)	624(18%)	3437
Did Not Participate	181(57%)	139(43%)	320
Maternal Reactions to Child’s Increased TIDM Risk			
Risk perception	N (%)	N (%)	N
Accurate	1809(84%)	355(16%)	2164
Underestimate	1132(77%)	343(23%)	1475
Data missing	53(45%)	65(55%)	118
State Anxiety Inventory score	M (SD)	M (SD)	M (SD)
Total Sample	38.7(9.7)	40.8(10.6)	39.1(9.9)
Risk Perception: Accurate	38.8(10.2)	41.7(10.4)	39.3(9.6)
Risk Perception: Underestimate	38.4(10.2)	39.9(10.8)	38.8(10.4)
	N (%)	N (%)	N
Data missing	46 (42%)	63 (58%)	109
Missing Data	N (%)	N (%)	N
≤1missing data points	2944 (81%)	695 (19%)	3639
>1 missing data points	50 (42%)	68 (58%)	118

**Table 3:** The 8 rules identified by the RuleFit method.

Rule 1 (risk increasing rule)	Rule 2 (risk increasing rule)
Maternal age <27.5 Finland = NO	Smoker during pregnancy = YES Accurate risk perception = NO State anxiety inventory score >45
Rule 3 (risk increasing rule)	Rule 4 (risk increasing rule)
State anxiety inventory score >45 Dad participation = NO	Maternal age <27.5 Accurate risk perception = NO Alcohol consumption in last trimester <2 times per month
Rule 5 (risk decreasing rule)	Rule 6 (risk decreasing rule)
Worked all trimesters = YES Smoker during pregnancy = NO	Finland = NO Alcohol consumption in last trimester >0 Number of negative events <2
Rule 7 (risk decreasing rule)	Rule 8 (risk decreasing rule)
Smoker during pregnancy = NO State anxiety inventory score < 45 Number of missing data points < = 1	Maternal age > 27.5 Smoker during pregnancy = NO Number of missing data points < = 1
