# Corrigendum: A Comparison of Rule-based Analysis with Regression Methods in Understanding the Risk Factors for Study Withdrawal in a Pediatric Study

**DOI:** 10.1038/srep39723

**Published:** 2017-05-02

**Authors:** Mona Haghighi, Suzanne Bennett Johnson, Xiaoning Qian, Kristian F. Lynch, Kendra Vehik, Shuai Huang, The TEDDY Study Group, Suzanne Bennett Johnson, Kristian F. Lynch, Kendra Vehik, Marian Rewers, Kimberly Bautista, Judith Baxter, Ruth Bedoy, Daniel Felipe-Morales, Kimberly Driscoll, Brigitte I. Frohnert, Patricia Gesualdo, Michelle Hoffman, Rachel Karban, Edwin Liu, Jill Norris, Adela Samper-Imaz, Andrea Steck, Kathleen Waugh, Hali Wright, Ashok Sharma, Diane Hopkins, Gabriela Young, Jin-Xiong She, Joshua Williams, Katherine Silvis, Leigh Steed, Melissa Gardiner, Richard McIndoe, Desmond Schatz, Jamie Thomas, Janey Adams, Laura Jacobsen, Michael Haller, Eric Triplett, Stephen W. Anderson, Juha Mykkänen, Katri Lindfors, Annika Adamsson, Sanna Jokipuu, Tiina Kallio, Leena Karlsson, Elina Mäntymäki, Petra Rajala, Mika Riikonen, Jenni Rouhiainen, Minna Romo, Maria Leppänen, Sini Vainionpää, Mari Vähä-Mäkilä, Aino Stenius, Jorma Toppari, Olli G. Simell, Tuula Simell, Maija Sjöberg, Eeva Varjonen, Heikki Hyöty, Mikael Knip, Kalle Kurppa, Maria Lönnrot, Tiina Niininen, Mia Nyblom, Suvi Ahonen, Lea Kovanen, Mirva Koreasalo, Anne Riikonen, Suvi M. Virtanen, Mari Åkerlund, Jorma Ilonen, Miia Kähönen, Tiina Latva-aho, Katja Multasuo, Riitta Veijola, Sari Niinistö, Jenna Rautanen, Anette G. Ziegler, Michael Hummel, Sandra Hummel, Nicole Janz, Annette Knopff, Claudia Peplow, Roswith Roth, Marlon Scholz, Joanna Stock, Katharina Warncke, Lorena Wendel, Christiane Winkler, Andreas Beyerlein, Ezio Bonifacio, Sibylle Koletzko, Kristina Foterek, Mathilde Kersting, Åke Lernmark, Daniel Agardh, Carin Andrén Aronsson, Maria Ask, Jenny Bremer, Ulla-Marie Carlsson, Corrado Cilio, Emelie Ericson-Hallström, Lina Fransson, Thomas Gard, Joanna Gerardsson, Rasmus Bennet, Monica Hansen, Gertie Hansson, Susanne Hyberg, Fredrik Johansen, Berglind Jonsdottir, Helena Elding Larsson, Marielle Lindström, Markus Lundgren, Maria Månsson-Martinez, Maria Markan, Jessica Melin, Zeliha Mestan, Karin Ottosson, Kobra Rahmati, Anita Ramelius, Falastin Salami, Sara Sibthorpe, Birgitta Sjöberg, Ulrica Swartling, Evelyn Tekum Amboh, Carina Törn, Anne Wallin, Åsa Wimar, Sofie Åberg, William A. Hagopian, Michael Killian, Claire Cowen Crouch, Jennifer Skidmore, Josephine Carson, Maria Dalzell, Kayleen Dunson, Rachel Hervey, Corbin Johnson, Rachel Lyons, Arlene Meyer, Denise Mulenga, Alexander Tarr, Morgan Uland, John Willis, Dorothy Becker, Margaret Franciscus, MaryEllen Dalmagro-Elias Smith, Ashi Daftary, Mary Beth Klein, Chrystal Yates, Jeffrey P. Krischer, Michael Abbondondolo, Sarah Austin-Gonzalez, Maryouri Avendano, Sandra Baethke, Rasheedah Brown, Brant Burkhardt, Martha Butterworth, Joanna Clasen, David Cuthbertson, Christopher Eberhard, Steven Fiske, Dena Garcia, Jennifer Garmeson, Veena Gowda, Kathleen Heyman, Francisco Perez Laras, Hye-Seung Lee, Shu Liu, Xiang Liu, Jamie Malloy, Cristina McCarthy, Steven Meulemans, Hemang Parikh, Chris Shaffer, Laura Smith, Susan Smith, Noah Sulman, Roy Tamura, Ulla Uusitalo, Ponni Vijayakandipan, Keith Wood, Jimin Yang, Beena Akolkar, Kasia Bourcier, Thomas Briese

Scientific Reports 6: Article number: 3082810.1038/srep30828; published online: 08 26 2016; updated: 05 02 2017

In the original version of this Article, the author list for the “TEDDY Study Group” was incomplete.

This has now been corrected in the PDF and HTML versions of this Article.

